# Association Between Nursing Diagnoses and Mortality in Hospitalized Patients with COVID-19: A Retrospective Cohort Study

**DOI:** 10.3390/nursrep15050147

**Published:** 2025-04-28

**Authors:** José Ángel Hernández-Mariano, Olivia Mendoza-Macario, María del Carmen Velázquez-Núñez, María del Carmen Cedillo-Ordaz, Blanca Estela Cervantes-Guzmán, Dulce Milagros Razo-Blanco-Hernández, Erick Alberto Landeros-Olvera, Fani Villa-Rivas, Rocío Castillo-Díaz, Guillermo Cano-Verdugo

**Affiliations:** 1 Department of Research, Hospital Juarez of Mexico, Mexico City 07760, Mexico; 2 Department of Quality and Health Education, Ministry of Health, Mexico City 11400, Mexico; 3 Head of Nursing Research, Hospital Juarez of Mexico, Mexico City 077602, Mexico; 4 Head of Resources Management for Nursing Care, Hospital Juarez of Mexico, Mexico City 077602, Mexico; 5 Nursing Directorate, Hospital Juarez of Mexico, Mexico City 077602, Mexico; 6 Faculty of Nursing, Meritorious Autonomous University of Puebla, Puebla 72410, Mexico; 7 Faculty of Nursing and Midwifery, Juarez University of the State of Durango, Durango 34217, Mexico; 8 School of Dentistry, Autonomous University of Nuevo Leon, Monterey 64460, Mexico

**Keywords:** COVID-19, mortality, nursing diagnoses, retrospective cohort study

## Abstract

Previous studies suggest that nursing diagnoses (NDs) could predict clinical outcomes, such as mortality, among patients with non-communicable diseases. However, evidence in patients with COVID-19 is still scarce. **Objective:** To evaluate the association between NDs and COVID-19 mortality among hospitalized patients. **Methods:** A retrospective cohort study was conducted on 498 paper clinical records of patients hospitalized for at least 72 h in the internal medicine unit for COVID-19 from June to December 2020. The interest association was assessed using logistic regression models. **Results:** NDs focused on COVID-19 pulmonary responses, such as impaired gas exchange (OR = 3.04; 95% CI = 1.87, 4.95), impaired spontaneous ventilation (OR = 3.67; 95% CI = 2.17, 6.21), or ineffective airway clearance (OR = 2.47; 95% CI = 1.48, 4.12), were significant predictors of mortality. NDs on COVID-19 extrapulmonary responses, such as risk for unstable blood glucose level (OR = 2.45; 95% CI = 1.45, 4,15), risk for impaired liver function (OR = 2.02; 95% CI = 1.11, 3.63), hyperthermia (OR = 2.08; 95% CI = 1.29, 3.35), decreased cardiac output (OR = 2.95; 95% CI = 1.42, 6.11), or risk for shock (OR = 3.03; 95% CI = 1.28, 7.13), were associated with a higher risk of in-hospital mortality. Conversely, patients with NDs of fear (OR = 0.56; 95% CI = 0.35, 0.89) and anxiety (OR = 0.44; 95% CI = 0.26, 0.77) had a lower risk of death. **Conclusions:** NDs on pulmonary and extrapulmonary responses to COVID-19 were associated with in-hospital mortality, suggesting that they are indicators of the severity of these patients. Therefore, NDs may help nursing staff identify individuals who require closer monitoring and guide early interventions for their recovery.

## 1. Introduction

SARS-CoV-2, the causative agent of COVID-19, emerged in December 2019, with the first reported cases in Wuhan, China. The virus rapidly spread across the globe, prompting the World Health Organization to declare COVID-19 a global pandemic on 11 March 2020 [1]. Although the severity of COVID-19 differs from person to person, most infected cases present mild to moderate symptoms with a good prognosis [2,3]. However, in 20% of unvaccinated cases, SARS-CoV-2 may cause severe lung disease and affect different organs, leading to multi-organ failure and death [4]. Thus, COVID-19 has had a catastrophic effect, resulting in approximately six million deaths worldwide, with the Americas being one of the most affected regions [5]. In 2020, 325,415 deaths associated with COVID-19 were reported in Mexico, which reduced the life expectancy at birth of Mexicans by 4.6 years between 2019 and 2020 [6].

Nurses are an integral part of the healthcare workforce, providing essential care to individuals, families, and communities. During the COVID-19 pandemic, nursing staff played a pivotal role in the health system’s response, serving on the front lines of hospital care and actively participating in patient assessment and monitoring within the community [7]. Nursing care involves problem-solving, critical thinking, and decision-making to attain the intended outcomes [8,9]. One of the main tools for developing the procedures nurses provide is the nursing care process, representing a practical model for providing evidence-based care. This approach promotes autonomy, creativity, and professional identity in nursing. The nursing process consists of five systematic phases: assessment, nursing diagnosis (ND), planning, implementation, and evaluation [10,11].

Nursing diagnoses (NDs) are the basis for the care plan of nurses and are different than medical diagnoses. They are clinical judgments about human responses to actual or potential health problems or needs based on a comprehensive assessment of subjective and objective data about the patient’s physical, psychological, sociocultural, and spiritual health [10,12]. Nurses have been incorporating a standardized language system to homogenize the terms used in patient care, allowing them to compare and evaluate the care provided in terms of effectiveness. Although other taxonomies exist, the NANDA-I (North American Nursing Diagnosis Association-International) is the best known and is available worldwide [13]. In Mexico, starting in 2007, the Permanent Commission on Nursing implemented strategies to promote standardized nursing language by using the NANDA-I, NIC, and NOC taxonomies in the Mexican health system. Therefore, nurses, nursing students, and educators are increasingly familiar with NANDA-I NDs. However, the practical application of these diagnoses in the clinical setting is still limited [14,15]. Previous studies have documented that nursing staff perceive workload as a significant barrier to implementing NDs in healthcare [16]. Moreover, the Mexican health system is fragmented into several health institutions, each with its regulations [14], which hinders the widespread application of NDs in all the country’s hospitals. Furthermore, nursing training programs differ among nursing schools across the country; therefore, proficiency in using NDs varies depending on the level of training. Unlike registered nurses, nursing assistants do not receive comprehensive training in standardized nursing language, although both often perform similar tasks [17].

The human responses identified through NDs are the individual’s reactions to the health-disease process; hence, they may reflect the severity of the patient’s illness, which correlates with a higher rate of complications, damage to vital organs, and an increased risk of mortality. This has been documented previously, where NDs on admission were associated with an increased risk of in-hospital mortality among patients with several chronic and surgical conditions [18,19,20,21,22,23]. Nevertheless, most previous studies have analyzed the association between the number of NDs and mortality but have not examined the individual effect of each diagnosis [18,19,20,23]. Besides, evidence in patients with emerging diseases, such as COVID-19, is still scarce. To our knowledge, only one study has evaluated the relationship between NDs and COVID-19 mortality [24]; hence, a knowledge gap remains. Even though the WHO announced that the COVID-19 epidemic no longer constitutes a health emergency of international concern, such disease remains a topic of interest in public health. Thus, identifying NDs associated with the highest mortality risk among COVID-19 patients may assist healthcare workers beyond nurses in guiding early interventions to improve patient outcomes. Furthermore, generating evidence on the impact of NDs in predicting patient outcomes could support their widespread use in nursing care in Mexican hospitals. Accordingly, we evaluated the association between NDs and mortality in hospitalized COVID-19 patients.

## 2. Materials and Methods

### 2.1. Design and Study Population

A retrospective cohort study was conducted at Hospital Juarez of Mexico (HJM), a tertiary-level hospital that is part of the Ministry of Health in Mexico City. HJM provides free, highly specialized medical care to people lacking social security, including medications and other related supplies. HJM users are mainly from the lower and lower-middle classes, primarily from central Mexico (Mexico City and the State of Mexico). A smaller proportion of patients come from other Mexican states. HJM has 433 hospitalization beds to serve approximately 16,000 visits annually.

During the COVID-19 pandemic, HJM was adapted into a COVID-19 Hospital; hence, some of its inpatient care services (i.e., internal medicine unit) were converted into intensive care units for the management of patients with severe COVID-19, and outpatient care and elective surgery services were suspended.

The inclusion criteria for the study were men and women aged 18 years or older who were hospitalized for at least 72 h in the internal medicine unit for COVID-19 from June to December 2020. Clinical records without nursing notes were excluded. Thus, 647 health records were initially reviewed, with 489 meeting the inclusion criteria and included in the analyses (75.5%).

### 2.2. Data Collection

Data were collected retrospectively by examination of paper clinical records, as electronic medical records are not used at HJM. For this purpose, we requested the medical records of patients with COVID-19 who were admitted to the intensive care unit between June and December 2020 from the medical records department. Due to concerns about poor inter-rater reliability, we conducted an independent exercise by extracting and reviewing 10% (n = 65) of the clinical records before applying the exclusion criteria. Cohen’s kappa coefficient [25], a statistic used to measure interobserver agreement, was 0.801, indicating a high degree of agreement with the research team’s decision on which cases to exclude.

Data from eligible clinical records were extracted and digitized directly into a database. We extracted the following information from each clinical record: patient outcomes (i.e., discharge or death), sociodemographic characteristics (i.e., age, sex, education, living status, occupation, and marital status), clinical variables at hospital admission (i.e., comorbidities, blood pressure levels, oxygen saturation, body temperature, and heart rate), and length of hospital stay.

NDs were obtained from nursing notes. It is essential to highlight that, since 2010, HJM has implemented the NANDA-I taxonomy for recording NDs, following the Mexican Standing Commission on Nursing recommendations. Therefore, nursing notes include a section where nurses handwrite each patient’s diagnoses. To assist with this, nursing staff can access the NANDA-I book for reference in each hospitalization unit. Besides, training courses on using NANDA-I taxonomy and the nursing care process are offered annually, but nurses are not required to take them.

### 2.3. Study Variables

Death due to COVID-19 was the primary outcome. The attending physician recorded the date of death. The NDs were independent variables. The labels of NDs reported at least once during each patient’s hospitalization were collected dichotomously (i.e., presence or absence). Therefore, to estimate the percentage of each nursing diagnosis, the number of patients who presented a certain nursing diagnosis at least once during their hospital stay was divided by the total number of patients included in this study. Finally, the covariates of interest were sociodemographic characteristics, clinical variables at hospital admission, and length of hospital stay. These covariates were used to identify potential confounders.

### 2.4. Statistical Analysis

The characteristics of the study sample and NDs were described using frequencies and percentages for categorical variables and medians with interquartile ranges for continuous variables. Normality was assessed using the Shapiro–Wilk test. Differences between survivors and non-survivors were analyzed using the two-tailed Pearson’s chi-square test for categorical variables and the two-tailed Mann–Whitney U test for continuous variables. The association between NDs and mortality among hospitalized COVID-19 patients was evaluated using logistic regression models for each identified ND. Odds ratios of mortality were estimated for patients with a particular ND, with those without the ND serving as the reference group. To evaluate the model’s goodness of fit, we used the Hosmer–Lemeshow test (*p* > 10, which indicates that the model fits the data satisfactorily).

All models were adjusted for confounding factors. The selection of potential confounders for inclusion in the model was based on directed acyclic graphs (DAGs) [26,27]. For this purpose, we used “DAGitty”, version 3.1(Deutsche Forschungsgemeinschaft, Germany), a free, browser-based tool for creatingDAGs [28]. The minimal sufficient set of variables for adjustment included age, sex, education, living status, and comorbidities (Supplementary Material S1).

Statistical significance for hypothesis tests and models was based on a *p*-value < 0.05. All analyses were performed using the STATA statistical package, version 15.1 (Stata Corporation, College Station, TX, USA).

## 3. Results

The data of 489 patients were analyzed, identifying the proportion of death at 39.5% (95% CI = 35.21, 43.88). The median age of the patients was 53 years, and most were men (54.6%). Compared to survivors, those who died were older, a higher percentage were men and retired workers, and a lower percentage had higher education (Table 1).

Regarding the patients’ clinical variables at hospital admission, we observed that the median oxygen saturation was 91%. One-third of the patients had an underlying medical condition (33.9%), and the median hospital stay was 11 days. The length of hospital stays, body temperature, and respiratory rate were higher in fatal cases (Table 2).

Twenty different nursing diagnoses were reported at least once during the hospital stay of the patients included in this study. The most prevalent nursing diagnoses focused on COVID-19 pulmonary and non-pulmonary human responses: ineffective breathing pattern, 92.6%; anxiety, 75.7%; impaired gas exchange, 51.3%; fear, 49.1%; hyperthermia, 47.4% (Table 3).

In fatal cases, a higher proportion of patients were diagnosed with risk for impaired liver function, risk for unstable blood glucose level, impaired gas exchange, decreased cardiac output, impaired spontaneous ventilation, risk for infection, risk for shock, risk for pressure injury in adults, adult pressure injury, ineffective airway clearance, and hyperthermia. In contrast, the survivor group had a lower proportion of patients diagnosed with fear and anxiety compared to non-survivors (Supplementary Material S2).

**Figure 1:**
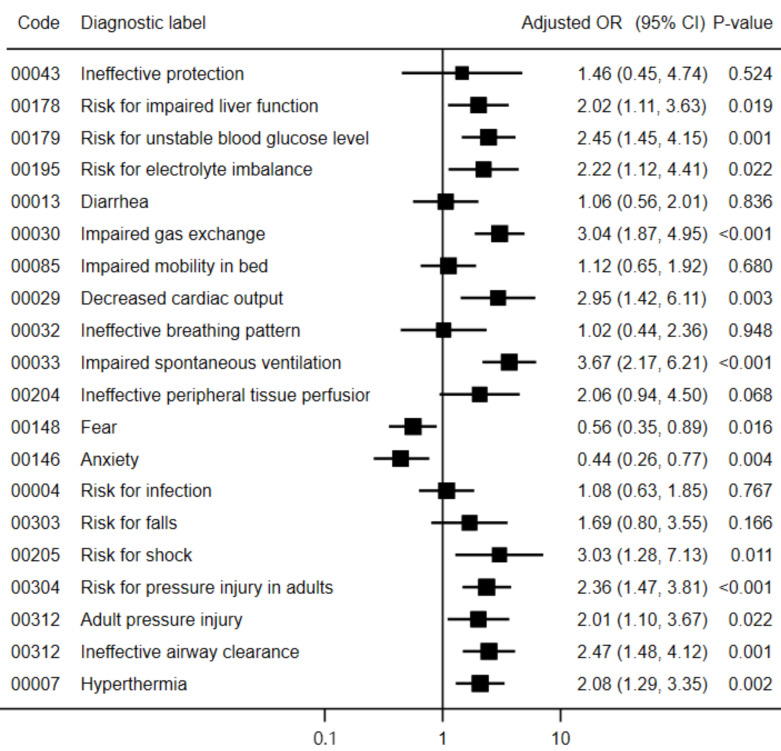
Adjusted odds ratio of the association between nursing diagnoses and mortality in patients with COVID-19. Abbreviations: OR, odds ratio; CI, confidence interval. According to the Hosmer–Lemeshow test, all models fit the data adequately (*p* > 0.10).

After adjustment for potential confounders, patients with NDs focused on COVID-19 pulmonary human responses, such as impaired gas exchange (adjusted odds ratio (aOR) = 3.04; 95% confidence interval = 1.87, 4.95), ineffective airway clearance (aOR = 2.47; 95% CI = 1.48, 4.12), and impaired spontaneous ventilation (aOR = 3.67; 95% CI = 2.17, 6.21), were associated with an increased risk of mortality. Moreover, patients with risk NDs for extrapulmonary human responses to COVID-19, such as adult pressure injury (aOR = 2.01; 95% CI = 1.10, 3.67) and hyperthermia (aOR = 2.08; 95% CI = 1.29, 3.35), were more likely to have a fatal outcome. Additionally, the risk NDs for extrapulmonary human responses to COVID-19, such as risk for impaired liver function (OR = 2.02; 95% CI = 1.11, 3,63), risk for unstable blood glucose level (aOR = 2.45; 95% CI = 1.45, 4.15), risk for electrolyte imbalance (aOR = 2.22; 95% CI = 1.12, 4,41), decreased cardiac output (aOR = 2.95; 95% CI = 1.42, 6.11), risk for shock (aOR = 3.03; 95% CI = 1.28, 7.13), and risk for pressure injury in adults (aOR = 2.36; 95% CI = 1.47, 3.81), were significant predictors of in-hospital mortality. On the other hand, patients with nursing diagnoses of fear (aOR = 0.56; 95% CI = 0.35, 0.89) and anxiety (aOR = 0.44; 95% CI = 0.26, 0.77) had a lower risk of death (Figure 1). Similar associations were observed in the crude models (Supplementary Material S3).

## 4. Discussion

In this study, NDs were significant predictors of in-hospital mortality in patients with COVID-19. Our findings reinforce the existing literature on the potential of NDs to predict patient outcomes. Given the limited research on standardized nursing language in the Mexican context, these results could support the nursing care processes in the country and, by extension, in the broader Latin American context, where the NANDA-I taxonomy is widely adopted.

The mortality rate reported in this study was higher compared to other countries, such as the United States, Italy, Denmark, and Spain, during the first year of the COVID-19 pandemic [29,30,31], but consistent with Latin American studies conducted in Peru, Colombia, and Brazil [32,33,34]. It has been proposed that pre-pandemic conditions, such as high informal employment, overcrowding, population density, health infrastructure, and social inequalities, may have played a significant role in the burden of COVID-19 mortality among Latin American patients [35,36].

SARS-CoV-2 infection can trigger a cytokine storm through hyperactivation of the immune system and the uncontrolled and elevated release of pro-inflammatory cytokines. Cytokine storms may cause acute respiratory distress syndrome (ARDS) and multi-organ failure, with a serious risk of death to the patient [37]. Patients with underlying conditions (i.e., obesity, hypertension, and diabetes) are at increased risk of cytokine storm and poor outcomes [38,39]. In our study population, 27% of patients suffered from underlying diseases, with hypertension being the most prevalent. These findings could explain the high COVID-19 mortality rate found.

To our knowledge, this is the first study in Mexico to evaluate the association between NDs and COVID-19 mortality and, overall, the first in the country to analyze the relationship between such diagnoses and clinical outcomes. Unlike most previous studies that relate the total number of diagnoses identified in a patient with in-hospital mortality [18,19,20,23], we evaluated the effect of each diagnosis. This approach is more informative because each diagnosis identifies specific human responses; therefore, the mechanism underlying its association with mortality could differ. Furthermore, this approach allows us to determine which diagnoses are associated with a higher or lower mortality risk.

Our findings showed that patients diagnosed with risk for impaired liver function had an increased risk of death. This ND refers to the existence of factors that raise the probability of an individual developing impaired liver function in the future. These results contrast with those reported by [24], who found a positive but not statistically significant association. However, in such a study, the sample size was smaller than ours, which could have limited their statistical power to detect a significant association. Liver injury has been a more frequent extrapulmonary manifestation among hospitalized patients with SARS-CoV-2 infection, and its presence has been associated with an increased risk of death [40]. SARS-CoV-2 appears to target cells that highly express the membrane protein angiotensin-converting enzyme 2. In the liver, there are receptors of angiotensin-converting enzyme 2, which contribute to the attachment and penetration of SARS-CoV-2 into hepatocytes, affecting the bile ducts [41]. Hence, the observed association in our study is biologically plausible.

Our results suggested that the ND of risk for unstable blood glucose levels was associated with COVID-19 mortality. This ND refers to a deviation from normal blood glucose levels that causes hyperglycemia or hypoglycemia. Unstable blood glucose is not only present in patients with diabetes. Different factors, such as dehydration or certain medications, can alter blood glucose levels. Overall, patients hospitalized with SARS-CoV-2 infection were treated with corticosteroid therapy. The anti-inflammatory action of corticosteroids can prevent and mitigate the cytokine storm effects, making them drugs capable of reducing the risk of ARDS and multi-organ failure in COVID-19 [42]. However, corticosteroids negatively affect glucose homeostasis, increasing blood glucose levels [43,44]. Furthermore, emerging evidence has suggested that COVID-19 disease may induce increases in serum glucose levels. Although the underlying mechanism has not been elucidated, it is now known that the SARS-CoV-2 virus can cause damage to the beta cells of the pancreas, which could explain the increase in glucose levels in patients with this infection [45].

We found that the ND of risk for electrolyte imbalance increased the odds of COVID-19 mortality. Risk for electrolyte imbalance refers to the possibility of alterations in serum electrolyte concentrations affecting health. Different studies have documented that SARS-CoV-2 infection is associated with electrolyte disturbances, resulting in progression to hospitalization, acute kidney injury, and 30-day mortality [46,47,48,49]. Patients with COVID-19 often present predominantly with hypokalemia, which may be due to increased urinary potassium excretion due to overactivation of the renin–angiotensin–aldosterone system [50]. Concern about hypokalemia is crucial due to the propensity of low potassium to perpetuate acute respiratory distress syndrome and arrhythmia [51].

We observed that those patients diagnosed with impaired gas exchange and impaired spontaneous ventilation were at higher risk of death compared to patients who did not have such NDs. These results agreed with those reported by [24], who also found positive associations. Impaired gas exchange refers to the presence of alterations in oxygenation or the elimination of carbon dioxide at the level of the alveolar-capillary membrane. In its severe form, COVID-19 causes acute respiratory distress syndrome, which is defined by the acute onset of noncardiogenic pulmonary edema, hypoxemia, and the need for mechanical ventilation. This disorder is associated with capillary endothelial injury and diffuse alveolar damage, therefore affecting the ability of the respiratory system to take in oxygen, and eliminate carbon dioxide [52,53].

Impaired spontaneous ventilation refers to the disability of patients to maintain spontaneous breathing, requiring ventilatory support. In most cases, it is caused by mechanical breathing problems, such as weakness of respiratory muscles. SARS-CoV-2 infection causes inflammatory damage to the lung parenchyma and decreases lung compliance, which may exacerbate the imbalance between breathing demands, and the force-generating capacity of respiratory muscles [54,55]. Furthermore, severe cases of COVID-19 requiring mechanical ventilation may experience rapid respiratory muscle atrophy and weakness [56]. Emerging evidence also suggests that SARS-CoV-2 infection could directly damage respiratory muscles [54]. In a postmortem study conducted in [57], myofiber membrane tissue from patients admitted to intensive care units with COVID-19 was compared with non-infected patients. They found that angiotensin-converting enzyme 2 was expressed in the myofiber membrane of the diaphragm and observed SARS-CoV-2 viral permeation into diaphragm myofibers among 26 patients who died from COVID-19. Moreover, a higher expression of genes associated with fibrosis was observed in patients with COVID-19, even though the duration of mechanical ventilation usage and length of hospital stay were similar to those without COVID-19 [57].

Ineffective airway clearance was associated with COVID-19 mortality. This ND is defined as the inability to clear secretions or obstructions from the respiratory tract to maintain a clear airway. Its main causes include respiratory tract infection, inflammation, mucus production, and airway obstruction. The airways of the lungs are lined with mucus-covered epithelial tissue that traps harmful pathogens and dust particles and then removes them by mucociliary clearance. The level of mucus in our body is regulated by mucus-secreting cells and mucociliary desquamation [58]. Nevertheless, the hypersecretion of mucus in respiratory conditions is associated with a sudden deterioration of lung function, an increased hospitalization rate, and mortality among affected individuals [59,60]. There is evidence linking mucus hypersecretion with COVID-19 disease severity [61]. As mentioned above, a severe SARS-CoV-2 infection may cause a cytokine storm. The proinflammatory cascades trigger the overproduction of mucus with altered composition and impaired mucociliary clearance in infected respiratory epithelia, resulting in increased airway obstruction and respiratory distress [62,63].

Decreased cardiac output is when the heart fails to pump an adequate volume of blood to meet the body’s metabolic demands. Its defining characteristics are categorized into five groups: heart rate/rhythm abnormality, preload abnormality, afterload abnormality, contractility abnormality, and behavioral/emotional abnormality. Conditions associated with this nursing diagnosis include abnormalities in contractility, heart rate, afterload, preload, heart rhythm, and stroke volume. Our study found that decreased cardiac output was associated with a higher mortality risk in COVID-19 patients. Several studies have similarly reported that COVID-19 infection can lead to a range of cardiac manifestations, including myocardial injury, myocarditis, acute coronary syndromes, heart failure, arrhythmias, and venous thromboembolism, all contributing to a poor prognosis [64,65,66,67].

Based on clinical and histological evidence, possible mechanisms of injury include direct viral insult or indirect damage to the heart due to hypoxemia because of acute respiratory distress syndrome, elevated thromboembolic risk, COVID-19-associated cytokine storm syndrome, and inflammation-mediated tissue injury [68,69,70,71].

In the present study, the ND of ineffective peripheral tissue perfusion was marginally associated with mortality in COVID-19 patients. A significant relationship between peripheral tissue perfusion and the severity of COVID-19 has been documented in previous studies. Patients with severe COVID-19 show systemic microcirculatory alterations suggestive of endothelial dysfunction, which are associated with the severity of acute respiratory distress syndrome [72,73].

Our analysis revealed that patients diagnosed with risk for shock had higher odds of death. Shock occurs in approximately 67% of hospitalized patients with COVID-19, and it has been associated with high mortality [74]. Most cases of shock are cardiogenic [75], but in other patients, shock may be septic [76]. Previous evidence suggests that SARS-CoV-2 may cause sepsis by itself, independently of secondary bacterial or fungal infections. Proposed mechanisms include immune dysregulation, respiratory dysfunction leading to hypoxemia, and metabolic acidosis due to circulatory dysfunction [76,77].

Risk of pressure injury in adults and pressure injury in adults were associated with an increased risk of death. Excessive hospitalization is a risk factor for pressure ulcers and COVID-19 mortality [78,79]. In our study, the median hospital stay among fatal cases was 13.5 days. Moreover, among hospitalized cases of COVID-19, prone positioning was an intervention to improve oxygenation parameters. However, one of the side effects of this intervention was the risk of pressure ulcers [80,81,82]. Therefore, the nursing diagnosis of risk of pressure injury in adults may be an indicator of the severity of COVID-19 patients, which could explain the association found.

Our findings revealed that the risk of death was higher among those patients diagnosed with hyperthermia. Previous studies have documented that elevated body temperature is associated with in-hospital mortality among patients with COVID-19 [83,84,85]. SARS-CoV-2 infection stimulates leukocytes to release cytokines, and in severely ill patients, a cytokine storm may be triggered, contributing to lymphopenia, lung injury, and multi-organ failure [86,87]. Of these cytokines, IL-6 is a major pyrogenic cytokine that elevates the core body temperature through autonomic thermoregulatory mechanisms and directly correlates with disease severity [88,89].

The results of the present study revealed that patients diagnosed with anxiety and fear had a lower risk of death. The ND refers to an emotional state that a person may experience when they feel a threat or danger, even if it is not specific or unknown. The second ND alludes to a basic and intense emotional response that arises when an imminent threat is detected. Besides physiological and cognitive manifestations, the defining characteristics of both diagnoses include the verbal manifestation of the patient’s feelings and emotions; thus, such diagnoses likely acted as indicator variables of the patient’s severity. The most severe cases of COVID-19 required ventilatory support [90]; hence, the ability of these patients to speak was limited. A systematic review developed in [91] documented that patients infected with SARS-CoV-2, who required ventilatory support, had a higher risk of death.

In contrast to the data reported by [24], we did not find an association between COVID-19 mortality and the ND of ineffective respiratory pattern. This ND refers to a state in which an individual’s inspiratory or expiratory pattern does not provide adequate ventilation. Dysfunctional breathing is a common manifestation of SARS-CoV-2 infection; hence, ineffective respiratory pattern has been one of the most common NDs in these patients [24,92,93]. In our study, the proportion of patients diagnosed with ineffective respiratory pattern was similar between those who died and those who survived, which could explain the null association observed.

Of our population, 11.4% received the ND of diarrhea. Angiotensin-converting enzyme 2, the entry receptor of SARS-CoV-2, is found in the stomach and small intestine; thus, there is a plausible basis for linking COVID-19 disease and gastrointestinal symptoms [94]. However, in our study, the proportion of patients with diarrhea was similar between survivors and fatal cases. Furthermore, we did not find a statistically significant association between both variables. These findings are consistent with a recent meta-analysis suggesting that diarrhea and other gastrointestinal symptoms are not associated with higher mortality of COVID-19 patients [95].

### 4.1. Limitations

Our results have some limitations that should be considered when interpreting the findings. Using a retrospective design may affect data quality; however, we anticipate that any recording errors were randomly distributed. Furthermore, due to the retrospective approach of this study, the estimated associations are not causal and should be interpreted with caution. Nonetheless, the identification of clinical records used in this study was based on COVID-19 disease and not mortality status; therefore, our results were unlikely to be affected by a selection bias. It is important to note that given the historical timeframe of this study, our patient cohort did not include individuals vaccinated against SARS-CoV-2, who currently constitute most of the population. While the severity of COVID-19 varies by individual, SARS-CoV-2 has been documented to cause severe lung disease in 20% of unvaccinated cases [4]. This may limit the generalizability of our findings to vaccinated patients. Thus, future research must be conducted on these patients and those with SARS-CoV-2 variants. In Mexico, nursing staff competency in using NDs varies depending on their level of training. Unlike registered nurses, nursing assistants do not receive comprehensive training in standardized nursing language, yet both often perform similar tasks. Therefore, we believe that critically ill patients were likely not exclusively cared for by registered nurses. If there were errors in NDs reporting, we expect them to have been randomly distributed. Finally, it is essential to highlight that the applicability of NANDA-I NDs to guide clinical practice has been strongly questioned by several authors who consider that they may lack clear evidence, are vague, or do not apply to real-world clinical situations [96,97,98,99,100,101,102,103]. Furthermore, some NANDA-I NDs may reflect U.S.-centric healthcare models, which do not always align with other countries’ practices, resources, or priorities. Nevertheless, the NANDA-I taxonomy is constantly updated to improve its proposed nursing diagnoses’ clarity, relevance, and scientific grounding. Hence, it is crucial to develop high-quality research on NANDA-I NDs that provides sufficient evidence to justify their clinical use and to adapt them to different healthcare settings.

### 4.2. Implications for Nursing Practice

The findings of this research could contribute to four key areas: nursing science from theoretical, practical, and educational perspectives, as well as to society. Given the scarcity of research on nursing diagnoses from a pragmatic viewpoint with pathophysiological support, these results demonstrated that the use of nursing diagnoses can help identify the risk of mortality when patients present with other health issues during hospitalization. This knowledge enables the establishment of elements necessary to develop timely and specialized care plans.

For nursing practice, it represents a means of communication between nursing colleagues and, at the same time, with members of other disciplines, mainly medicine. This situation can improve the quality of nursing care and the effectiveness of pharmacological treatment.

The contribution to the educational field shortens the distance between theory and practice. At least for Mexico and the Latin American context, it represents evidence so that study plans can present content of the usefulness of nursing diagnoses (and nursing language in general) that continues to be taught in the classroom, but without being reflected in the reality of nursing services in the hospitals.

Likewise, the findings contribute to society, since they contribute to the epidemiological knowledge of COVID-19, and they can contribute to containing mortality by acting in advance on specific or specialized nursing care. Therefore, the findings indirectly allow families to reunite after the patient recovers. Likewise, each of these diagnoses represents a nursing care continuity plan at home, which must be executed by the family members and the patient themselves so that they can achieve self-care.

## 5. Conclusions

The results of our study showed that NANDA-I NDs are independent predictors of mortality in patients with COVID-19; hence, their use could contribute to the timely identification of the risk of death among hospitalized patients with severe acute respiratory infection and guide specialized care plans focused on the recovery of these patients. Since few studies have evaluated the individual effect of NANDA-I NDs on patient outcomes, such as mortality, it is essential to develop high-quality research that allows us to confirm or refute our findings and to address their biological and clinical relevance.

## Figures and Tables

**Table 1:** General characteristics of the study population according to mortality status.

Characteristics	Total (N = 489)	Survivors N = 296 (60.5%)	Deaths N = 193 (39.5%)	*p*-Value ^a^
Sex, f (%)				
Women	222 (45.4)	151 (51.1)	71 (36.8)	0.002
Men	267 (54.6)	145 (58.9)	122 (63.2)	
Age (in years)				
Median (IQR)	53 (18)	47 (16)	63 (15)	<0.001
Marital status, f (%)				
Unmarried	155 (31.7)	92 (31.1)	63 (32.6)	0.717
Married	334 (68.3)	204 (68.9)	130 (67.4)	
Educational level, f (%)				
No education	52 (10.6)	31 (10.5)	21 (10.9)	0.009
Basic education	135 (27.6)	66 (22.3)	69 (35.8)	
Intermediate level	190 (38.9)	127 (42.9)	63 (32.6)	
Higher education	112 (22.9)	72 (24.3)	40 (20.7)	
Employment status, f (%)				
Paid work	296 (60.5)	177 (59.8)	119 (61.7)	0.001
Retired	43 (8.8)	16 (5.4)	27 (14.0)	
Other	150 (38.9)	103 (34.8)	47 (24.3)	

Abbreviations: f, frequency; IQR, interquartile range. ^a^ Comparing subjects by COVID-19 mortality status using Pearson’s chi-squared test for categorical variables and the Mann–Whitney U test for the difference in medians.

**Table 2:** Clinical characteristics of patients at hospital admission according to mortality status.

Clinical Characteristics	Total (N = 489)	Survivors N = 296 (60.5%)	Deaths N = 193 (39.5%)	*p*-Value ^a^
SBP (mmHg)				
Median (IQR)	120 (10)	120 (20)	124 (20)	0.053
DPB (mmHg)				
Median (IQR)	80 (12)	78 (10)	80 (14)	0.057
Body temperature (degrees Celsius)				
Median (IQR)	37.5 (1.8)	37.4 (1.4)	38.0 (0.9)	0.001
Heart rate				
Median (IQR)	80 (18)	80 (18)	82 (18)	0.067
Respiratory rate				
Median (IQR)	24 (6)	22. (5)	24 (7)	<0.001
Oxygen saturation				
Median (IQR)	91 (3)	92 (4)	90 (4)	0.002
Comorbidities, f (%)				
No	323 (66.1)	220 (74.3)	103 (53.4)	<0.001
Yes	166 (33.9)	76 (25.7)	90 (46.6)	
Length of hospital stay				
Median (IQR)	11 (8)	10 (6.5)	13.5 (12)	0.002

Abbreviations: SBP, systolic blood pressure; DBP, diastolic blood pressure; mmHg, millimeter of mercury; f, frequency; IQR, interquartile range. ^a^ Comparing subjects by COVID-19 mortality status using Pearson’s chi-squared test for categorical variables and the Mann–Whitney U test for the difference in medians.

**Table 3:** Nursing diagnoses by domains and classes.

Domains	Class and Diagnostic Label (Code)	Total (N = 489)	
		f	%
DOMAIN 1. Health promotion	Class 2. Health management		
	(00043) Ineffective protection	19	3.9
DOMAIN 2. Nutrition	Class 4. Metabolism		
	(00178) Risk of impaired liver function	88	18.0
	(00179) Risk for unstable blood glucose level	131	26.8
	Class 5. Hydration		
	(00195) Risk for electrolyte imbalance	63	12.8
DOMAIN 3. Elimination and exchange	Class 2. Gastrointestinal function		
	(00013) Diarrhea	74	15.1
	Class 4. Respiratory function		
	(00030) Impaired gas exchange	251	51.3
	Class 2. Activity/Exercise		
	(00085) Impaired physical mobility	119	24.3
DOMAIN 4. Activity/Exercise	Class 4. Cardiovascular-pulmonary responses		
	(00029) Decreased cardiac output	58	11.9
	(00032) Ineffective breathing pattern	453	92.6
	(00033) Impaired spontaneous ventilation	137.	28.0
	(00204) Ineffective peripheral tissue perfusion	48	9.8
DOMAIN 9. Coping/stress tolerance	Class 2. Coping responses		
	(00148) Fear	240	49.1
	(00146) Anxiety	370	75.7
DOMAIN 11. Safety/Protection	Class 1. Infection		
	(00004) Risk of infection	116	23.7
	Class 2. Physical injury		
	(00303) Risk for falls	44	9.8
	(00205) Risk for shock	40	8.2
	(00304) Risk for pressure injury in adults	182	37.2
	(00312) Adult pressure injury	87	17.9
	(00031) Ineffective airway clearance	141	28.8
	Class 4. Thermoregulation		
	(00007) Hyperthermia	232	47.4

Abbreviations: f, frequency.

## Data Availability

The data that support the findings of this study are openly available in Mendeley Data at http://doi.org/10.17632/rshxkkxb37.1, Accessed on 7 November 2024.

## Supplementary Materials

The following supporting information can be downloaded at: https://www.mdpi.com/article/10.3390/nursrep15050147/s1. Supplementary Material S1: Directed acyclic graph for the association between nursing diagnoses and COVID-19 mortality. Supplementary Material S2: Nursing diagnoses according to mortality status. Supplementary Material S3: Crude odds ratios of the association between nursing diagnoses and mortality in patients with COVID-19.

## Public Involvement Statement

There was no public involvement in any aspect of this research.

## Guidelines and Standards Statement

This manuscript was drafted against the Strengthening the Reporting of Observational Studies in Epidemiology (STROBE) checklist for cohort research.

## Abbreviations

The following abbreviations are used in this manuscript:

ND	Nursing diagnosis
NDs	Nursing diagnoses
HJM	Hospital Juarez of Mexico
DAGs	Directed acyclic graphs
OR	Odds ratio
aOR	Adjusted odds ratio
IQR	Interquartile range
