# Supplementary material for: Association Between Nursing Diagnoses and Mortality in Hospitalized Patients with COVID-19: A Retrospective Cohort Study
Source: Nurs Rep. 2025 Apr 28;15(5):147. doi: 10.3390/nursrep15050147 (PMC12114455; doi:10.3390/nursrep15050147)
Supplement: Supplementary file 1 [file nursrep-15-00147-s001.zip › Supplementary Material S1.pdf]

Supplementary Material S1. Directed acyclic graph for the association between nursing diagnoses and COVID-19 mortality

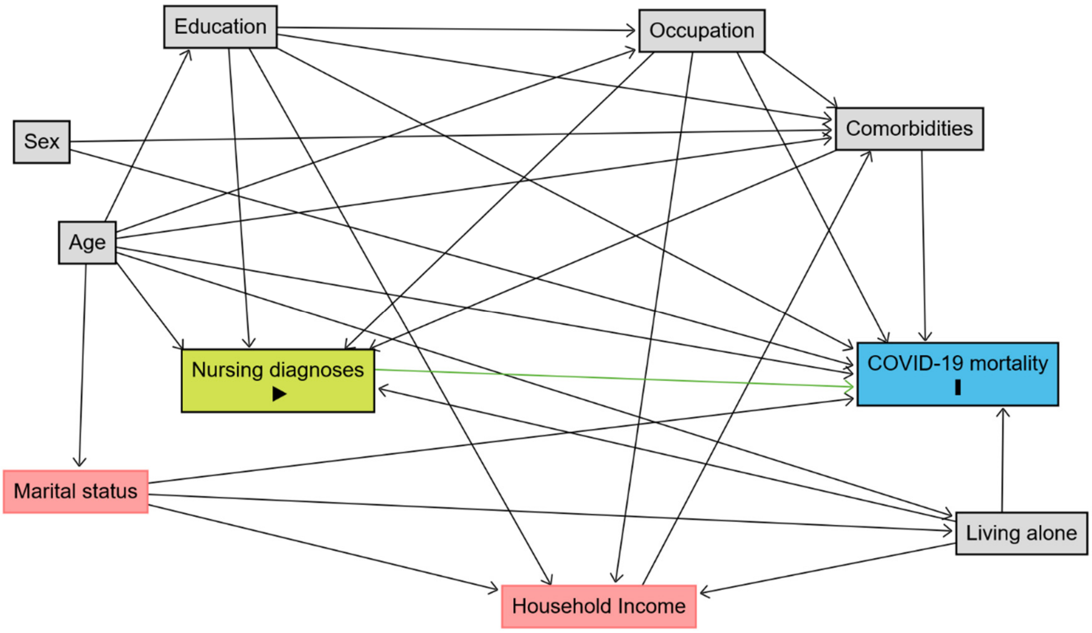

This directed acyclic graph was created in "DAGitty" [28]
