# Supplementary material for: Association Between Nursing Diagnoses and Mortality in Hospitalized Patients with COVID-19: A Retrospective Cohort Study
Source: Nurs Rep. 2025 Apr 28;15(5):147. doi: 10.3390/nursrep15050147 (PMC12114455; doi:10.3390/nursrep15050147)
Supplement: Supplementary file 1 [file nursrep-15-00147-s001.zip › Supplementary Material S2.pdf]

Supplementary Material S2. Nursing diagnoses according to mortality status

Nursing Diagnosis	Total (n=489)	Survivors n=296 (60.5%)	Deaths n=193 (39.5%)	Valor p[a]
**DOMAIN 1. Health promotion**				
Ineffective protection	19 (3.9)	8 (2.7)	11 (5.7)	0.094
**DOMAIN 1. Nutrition**				
Risk for impaired liver function	88 (18.0)	43 (14.5)	45 (23.2)	0.013
Risk of unstable blood glucose level	131 (26.8)	41 (13.9)	90 (46.6)	<0.001
Risk for electrolyte imbalance	63 (12.8)	30 (10.5)	33 (17.10)	0.025
**DOMAIN 3. Elimination and exchange**				
Diarrhea	74 (15.1)	45 (15.2)	29 (15.0)	0.957
Impaired gas exchange	445 (51.3)	106 (35.8)	145 (75.1)	<0.001
Impaired physical mobility	119 (24.3)	63 (21.3)	56 (29.0)	0.051
DOMAIN 4. Activity/rest				
Decreased cardiac output	58 (11.9)	23 (7.8)	35 (18.1)	0.001
Ineffective breathing pattern	453 (92.6)	276 (93.2)	177 (91.7)	0.526
Impaired spontaneous ventilation	137 (28.0)	55 (18.6)	82 (42.5)	<0.001
Ineffective peripheral tissue perfusion	48 (9.8)	17 (5.7)	31 (16.1)	0.001
DOMAIN 9. Coping/Stress tolerance				
Fear	240 (49.1)	172 (58.1)	68 (35.2)	<0.001
Anxiety	370 (75.7)	250 (84.5)	120 (62.2)	<0.001
DOMAIN 11. Safety/Protection				
Risk for infection	116 (23.7)	61 (20.6)	55 (28.5)	0.045
Risk for falls	48 (9.8)	25 (8.5)	23 (11.9)	0.207
Risk for shock	40 (8.2)	14 (4.7)	26 (13,5)	0.001
Risk for pressure injury in adults	182 (37.2)	77 (26.0)	105 (54.4)	<0.001
Adults pressure injury	87 (17.9)	42 (14.2)	45 (23.2)	0.010
Ineffective airway clearance	141 (28.8)	62 (20.9)	79 (40,9)	<0.001
Hyperthermia	232 (47.4)	105 (35.5)	127 (65.8)	<0.001

[a] Comparing subjects by COVID-19 mortality status using Pearson's chi-squared test for categorical variables
