# Supplementary material for: Association Between Nursing Diagnoses and Mortality in Hospitalized Patients with COVID-19: A Retrospective Cohort Study
Source: Nurs Rep. 2025 Apr 28;15(5):147. doi: 10.3390/nursrep15050147 (PMC12114455; doi:10.3390/nursrep15050147)
Supplement: Supplementary file 1 [file nursrep-15-00147-s001.zip › Supplementary Material S3.pdf]

Supplementary Material S3. Crude odds ratio of the association between Nursing Diagnoses and mortality in patients with COVID-19.

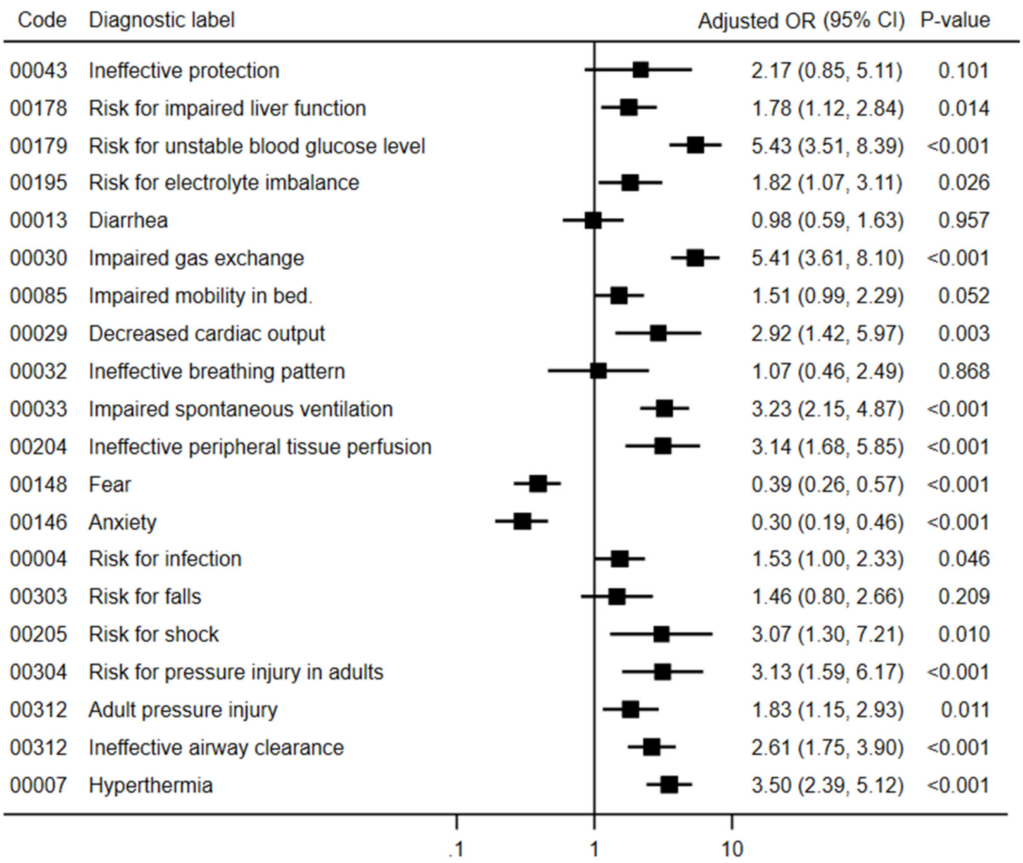

Abbreviations: OR, odds ratio; CI, Confidence Interval

According to the Hosmer-Lemeshow test, all models fit the data adequately (p >.10)
